# Melatonin and Abeta, Macular Degeneration and Alzheimers Disease: Same Disease, Different Outcomes?

**Published:** 2012

**Authors:** Bajic Vladan, Isabella Panfoli

**Affiliations:** 1 Institute for Pharmaceutical Research and Development, & University of Belgrade, Belgrade, Serbia; 2 Department of Pharmacy, University of Genoa, Genova, Italy

**Keywords:** Malatonin, Age related macular degeneration, Alzheimers

## Abstract

Aging is the common denominator and the highest risk factor for macular degeneration and Alzheimers Disease (AD). Important pathological hallmarks common to both diseases are the presence of amyloid β (Aβ) in the senile plaques of the AD brain and in the drusen of age-related macular degeneration (AMD) patients, oxidative stress, and apoptotic cell death. Data suggest that a common pathogenic mechanism might exist between AMD and AD. Brain and eye depend on redox electrons from pyridinic and flavinic nucleotides to produce ATP, and reactive oxygen intermediates (ROI). Disorganization of mitochondrial structure and decline in mitochondrial oxidative phosphorylation (OXPHOS) functioning, as well as hypometabolism and alterations in mitochondrial DNA are aging features. Because ROI damage and mitochondrial dysregulation are prominent in AMD and AD and their relationship to the redox state is unclear we addressed a new hypothesis according to which the interaction of melatonin vs Aβ are intertwined to balance of the intra- and extra-mitochondrial energy production. This balance would be impaired by the ageing process and environmental/genetic factors, ultimately leading to AD and /or AMD.

## INTRODUCTION

Aging is a common risk factor in both age-related macular degeneration (AMD) and Alzheimer's disease (AD). The incidence of AMD is increasing in the aging population. The World Health Organization has stated that AMD is the most common form of blindness, with 1.75 million people in the US alone and 7 million people at risk   [1]. AD is the most common dementia, doubling every 6 years after the age of 65. In Western countries, AD affects 1–3% of people aged 60–64 years, and 3–12% of people aged 70–80 years. It is estimated that by the mid-century (2050) as much as 13,2 million people will be affected by AD in the US alone   [2]. At the molecular level the pathognostic feature of AD is the accumulation of the 39-4 amino acid long β-Amyloid (Aβ) peptide with more that 50% of autopsy cases showing positive correlation. Aβ is also deposited in Drusen in AMD   [3]. A prospective population-based Rotterdam Study found that the neuronal degeneration occurring in AMD and AD may, to some extent, represent an evidence of a possible epidemiological connection between the two diseases, but with different origin as for genetic risks [4]. Interestingly, there is a slight prevalence of the female gender towards AMD   [5] and AD   [6,7]. AMD and AD appear linked because the retina is part of the brain [8], deriving from the neural tube which is the precursor of CNS development; moreover, both have blood–tissue barriers. At present, chronic oxidative stress, inflammation and altered fatty acid metabolism are strongly linked to AMD   [9**,**^10^] and also to AD pathogenesis   [11,12,13,14]. 

**Figure 1:**
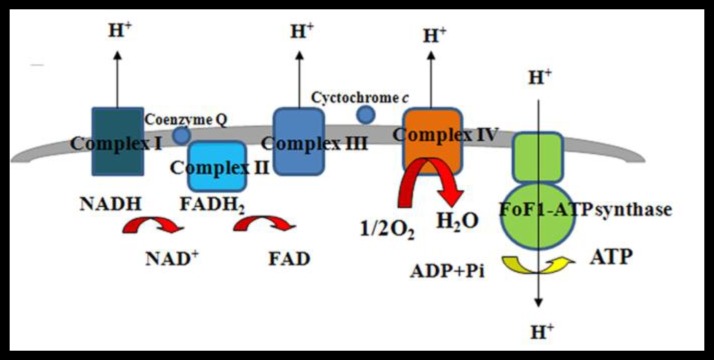
A schematic of the electron transport chain occurring in the inner mitochondrial membrane

**Aging and the mitochondrion**

CNS and retina critically depend on oxygen (O_2_) supply [15]*, *[16 ] and are sensitive to mitochondrial dysfunction    [17 ]. However, mitochondrial disorders, human diseases characterized by genetic defects of the oxidative phosphorylation (OXPHOS), affect the visual and the nervous system, even though these display a relative scarcity of mitochondria [17] . Mitochondrial dysfunctions are involved in pathologies associated with many diseases, such as, cancer, neurodegenerative diseases and aging. Aging is an incompletely understood process, in which a decline in mitochondrial function seems to be involved [18]. 

Mitochondria display two membranes, the outer membrane allowing the passage of low molecular-weight substances thanks to porin expression, [19], and the inner membrane (IM) housing the electron transfer chain (ETC) and providing a highly efficient barrier to the ion flow. The IM forms invaginations called *cristae* where the ETC complexes I-IV are embedded. These enable the transfer of electrons from NADH and FADH synthesized by glycolysis, fatty acid oxidation and Tricarboxylic acid cycle (TCA) to reduce molecular O_2_ to water [20]. During electron transfer, energy is used to pump protons in the intra membrane space, which promotes ATP generation via OXPHOS, thanks to the nanomotor ATP synthase (complex V). Proton gradient generates a chemiosmotic proton potential driving ADP phosphorylation of ADP to ATP (Figure 1).

In yeast, oligomeric organization of ATP synthase was reported to be essential to the maintenance of the mitochondrial *cristae* architecture and to correlate with maximum energy conversion capability [21], with an age-associated decline in ATP synthase oligomers. Prior data   [20,22,23,24]  suggest that the electron transport chain (ETC) and F_1_F_o_-ATP synthase are functionally expressed in extramitochondrial locations of the central nervous system, i.e: rod outer segment (OS) disks and isolated myelin vesicles [  22, 23, 24]. While the mitochondrial proteome consists of more than 1,000 different proteins, many proteomic analyses of cellular membranes have found the exclusive expression of proteins from the five respiratory complexes [ (reviewed in Panfoli e al. (25)]. Moreover, the enzymes of the Tricarboxylic Acid (TCA) Cycle are catalytically active in the rod outer segment [20 ], in keeping with the knowledge that many mitochondrial proteins possess dual or multiple localization [26] and that mitochondria are dynamic organelles, [27] . E*x vivo* staining of the optic nerve and retina   [22,23]  with MitoTracker (MT), a fluorescent mitochondrial probe sensitive to proton potential , showed that a proton potential is present in rod OS [28]. Mitochondria are currently believed to be central to a both life and death processes, such as energy production, and generation of reactive oxygen intermediates (ROI). However ROI would also be generated by the ectopic ETC coupled to ATP synthase. In fact an ETC not adequately coupled may generate ROI in turn oxidizing the polyunsaturated fatty acids of which the rod OS is rich.

Aging is consistently related to oxidative damage of cellular macromolecules due to ROI production [26, 29]. Impairment in mitochondrial OXPHOS functioning, alterations in mitochondrial DNA (mtDNA), increased production of ROI, with disorganization of mitochondrial structure have been reported with aging [30 ]. During the electron transfer 0.4% to 5% of ETC participate in the formation of superoxide radicals (O_2_^•-^) [31], therefore ROI are a physiological by-product of the ETC. However, an increase in O_2_^•-^ production can activate the mitochondrial permeability transition pore [32], ultimately committing cell to death by apoptosis. A study by Ghosh et al [.] showed that a redox shift precedes ROI changes in 3xTg-AD mice, i.e. a more oxidized redox state and a lower antioxidant GSH defense precedes neuronal damage, and the onset of cognitive defects. This means that even before cells accumulate harmful free radicals, they have changes in their reduction-oxidation reactions (redox). These results would explain why synapses go haywire long before people with Alzheimer’s disease experience any problems with memory [13]. The findings that the “ redox shift precedes ROI changes‘’ in AD mice directly points mostly to our hypothesis showing that the melatonin-Aβaxis may alter mitochondrial energy balance during aging leading to AMD and or AD. Mitochondrial DNA polymorphisms that augment ATP production can reduce Aβ load in mice [34]. It was reported that mitochondrial DNA (mtDNA) mutations can promote aging also independently of enhanced ROI production [35] accumulation of mutations in mtDNA [35]. These were in turn associated to reduced life span, and to aging signs.

In aging the ETC enzyme activity decrease, along with mitochondrial membrane potential. Parallely mitochondrial proteins and mtDNA are oxidatively damaged and there is a quantitative increase in mtDNA mutations. For example, Liang FQ et al., 2003 [36] showed that, when exposed to H_2_O_2,_ human retinal pigmented epithelium (RPE) cells or rod outer segments display mtDNA but not nDNA damage. Authors concluded that the susceptibility of mtDNA to oxidative damage, and decreased anti-oxidant system capability provides a rationale for mitochondria based model of AMD [37, 38]. Using the same rationale Liang FQ et a., 2004 [37] observed that RPE cells pretreated with melatonin show a significant decrease in mtDNA damage. Another pathway to mitochondrial damage is through the action of oligomeric Aβ to induce alterations of intracellular Ca(2+) levels and to promote the excess accumulation of intracellular Ca(2+) into mitochondria, thus inducing the mitochondrial permeability transition pore opening [31]. Increasing evidence suggests that the amyloid precursor protein (APP) and Aβ accumulate in mitochondrial membranes, cause mitochondrial structural and functional damage by generating ROI, hindering normal neuronal functioning [39,40]. Inhibition of ATP synthase inhibits the electron transport and OXPHOS. Such inhibition can be induced by Aβ [41]. Rhein et al, 2009 [42] reported that Aβ also lead to impaired functions of the mitochondria in human neuroblastoma cells. 

**Drusen and Amyloid plaques, different but the same?**

Extracellular protein deposits called drusen, accumulating between the RPE and photoreceptors, are a typical feature of non-neovascular AMD [43]. Drusen area and size positively correlate to risk of AMD progression [44]. Drusen are composed of acute phase proteins, complement components, proteglycans, apolipoproteins, metal ions (Fe, Zn, Cu), proteases ,cholinesterases, lipids [16,17,22], polysaccharides and ATP synthase subunit β [45] Some of these components are made by the eye itself, i.e. retina, RPE and/or choroid [46]. Wang and Wang [47] showed that the most abundant molecules in Drusen where esterified cholesterol and phosphatidine choline which suggest abnormalities in the metabolism of cholesterol, a risk factor also in AD [48]. Isas et al, 2010 [49], found that among the amyloid forms (oligomers, protofibrils, fibrils) the non-fibrilar oligomers where the most abundant form of amyloid in Drusen. Recently, amyloid vesicles as forms pervading in Drusen have also been reported in brains [50] of transgenic mice expressing human APP, suggesting the importance of APP processing in both eye and brain. Aβ accumulation has also been demonstrated in association with drusen in eyes from AMD patients [51, 52, 53] mice models for AMD [50] and in RPE [3]. Recently, Barrett et al., 2012 [54] showed that cholesterol directly binds to the C99 fragment of APP. This fragment, the result of β-secretase cleavage, is important for AD pathology because it is cleaved by γ-secretase to release Aβ. 

A causative role of oxidative stress and light exposure in the pathogenesis of AMD and other retinal degeneration has also been proposed [55 ] [56 ] [57]  [58]  [59] [60]. A critical role of SOD1 in protecting from AMD has been reported [61]. The choroid and retina are the highest oxygen-consuming tissues in the human body. The OS expressing oxygen-absorbing cytochrome *c* oxidase [22], would be at risk of oxidative stress oxidizing disk membranes, that contain high levels of polyunsaturated fatty acids. ROI are in fact a by-product of the ETC   [17]      [62] [63]. The result may be photoreceptor loss and visual impairment [64]. Inflammatory responses secondary to oxidative stress have been involved in age-related degenerative diseases. Oxidative stress induces the assembly of inflammatory protein complexes, the so-called inflammasomes, involving nod-like receptor protein 3 (NLRP3) [12]. The inflammasomes recognize danger signals, such as metabolic stress from ROI production, triggering inflammatory responses [12]. It was reported that misfolded protein aggregates such as amyloid-β can trigger NLRP3 inflammasome representing a pathogenetic mechanism in AD. Damaged mitochondria undergo digestion through mitophagy, a specialized form of autophagy, whose impairment may cause aging [65]. Autophagic capacity seems to be compromised in AD [66] and AMD [67]. Melatonin exerting its activity on Aβ in inflammation was presented by the work of Zhou et al. [68.] who found that microglia. i.e. the phagocytes of the nervous system, decrease superoxide anion production by impairing NADPH oxidase assembly in cultures of microglia. 

**APP/Aβ metabolism in the Eye and Brain**

Characteristic pathological features of AD are cerebral plaques with *β*-amyloid peptide and neurofibrillary tangles. However, as Aβ and tangles appear a normal finding in brains of non-demented individuals, these may be related to brain aging, independently of AD, suggesting their wider hypometabolic origin. The 2011 AD criteria proposes the presence of low CSF Aβ and decreased glucose utilization as AD biomarkers. Aβ is in small amount deposited in the brain [69] and in normal retina [51, 70] and the levels of these deposits increase during aging [71]. Johnson et al. [51] where the first to propose the pathogenic role of Aβ in AMD. Activated component complement component of RPE deposits where co localized to Aβ detected by using immunohistochemical technique. It was shown that Aβ can be detected in sub RPE basal deposits and neurovascular lesions in murine model of AMD [72]. Accumulation of Aβ in the eye occurs primarily among the photoreceptor OS and in the interphase between the RPE and Bruch’s membrane. Indeed, an origin of drusen in OS has never been supposed, but considering their ability to manipulate O_2_ should be taken into consideration. Such accumulation of Aβ on photoreceptor outer segments with age was confirmed in human retina using immunohistochemistry [71]. This implies that the accumulation of Aβ is associated with efficiency of RPE phagocytic process [3], but also through APP metabolism [73]. Sarangarajan and Apte [74] showed that signaling pathways that upregulate melanization in the RPE may be implicated in down-regulation of the rod OS phagocytosis by RPE, maintaining a balance between ingestion and degradation/recycling lowering metabolic load, suggesting a possible Aβ vs melatonin/melanin interaction in the balance of mitochondrial energy metabolism. Yoshida et al. [75] showed that human RPE expresses constitutively all of the genes that regulate Aβ production ,e.g., APP,α ,β,γ secretase and neprylisin. 

Melanization activating pathways may also modulate O_2_ consumption by the photoreceptors, and the rate of photoisomerization events such that the net effect may be a reduction in drusen and/or lipofuscin accumulation. This interaction may play a role in decreasing choroidal neovascularization. The hormone melatonin may have regulatory effects on APP metabolism. Interestingly, melatonin plays a fundamental role in retinogensis through APP metabolism [10,73,75]. Cultured RPE cells exposed to Aβ increase the expression of VEGF and decrease Pigment Epithelium-derived factor (PEDF, a potent antiangiogenic factor). Balance between these two molecules are important for healthy retina [76]. 

Melatonin treatment inhibited normal levels of secretion of soluble APP (sAPP) in different cell lines by interfering with APP full maturation [77]. Melatonin also affects the mRNA level of APP in a cell type-specific manner. Additionally, administration of melatonin efficiently reduced Aβ generation and deposition both *in vivo* [78, 79] and *in vitro* [77]. Moreover, it has been reported that mitochondrial dysfunction is characteristic of A*β*-induced neuronal toxicity in AD. A mitochondrial cascade hypothesis was proposed postulating that A*β* production, and tau phosphorylation, are consequences of impaired mitochondrial function and hypometabolism. Interestingly, the activity of mitochondrial enzymes (such as pyruvate- and ketoglutarate-dehydrogenase) as well as of some respiratory complexes (NADH:ubiquinone oxidoreductase, complex I, and cytochrome oxidase; complex IV, both partly coded by mitochondrial DNA) are reduced in mitochondria from AD subjects. 

## HYPOTHESIS

Considerng the findings of Panfoli et al., [25, 60] and others [42, 31, 41, 80], the present paper proposes the hypothesis of a role for melatonin-Aβ axis in mitochondria, and that the interaction of melatonin vs Aβ are intertwined to the balance of the inter and extra mitochondrial energy production. This balance would be deregulated by the ageing process and other environmental/genetic factors, in turn leading to hypometabolism and neurodegenerative diseases characterized by protein deposition, such as AD and /or AMD.

**Evaluation and Disscussion of the Hypothesis**

Cumulative oxidative status plays a critical role to AMD and AD, both age related disorders [61]. A large gradient of oxygen towards the inner retina [81] fits with an extra mitochondrial respiration [22]. Panfoli et al. [60] proposed a bioenergetic hypothesis drusen, which may originate through hypometabolism , in turn imbalancing clearance of proteins causing aggregation of peptides that accumulate [60]. In fact the OS, that contains high levels of polyunsaturated fatty acids and expresses oxygen-absorbing OXPHOS machinery   [82] , outside mitochondria, is at risk of oxidative stress. ROI are in fact a by-product of the ETC [17]   [62] [63]. ROI in turn may cause damage to RPE, increase the production of VEGF (Vascular Endothelial Growth Factor). Interestingly, Biochemical and histochemical analyses demonstrated that the labeled protein accumulating in the cytosol of Alzheimer degenerating neurons is the α-chain of the ATP synthase [83 ]. It is specifically observed in degenerating neurons, either alone or tightly associated with aggregates of tau proteins, suggesting that it is a new molecular event related to neurodegeneration. This may be the initiating factor in retinal degenerative diseases, but also in AD, both characterized by extracellular deposits of proteins. Extensive literature demonstrate melatonin antioxidant capacity [84 and refs. therein] both *in vivo* and *in vitro*. Its major action is maintenance of mitochondrial protein homeostasis.

 Interestingly, a modified model of the mitochondrial hypothesis for AD has been proposed, in which A*β *would cause neurotoxicity by interacting with mitochondrial targets or being itself intramitochondrial [85 ]. To strengthen the extramitochondrial idea, Schmidt et al. [86] showed in vitro that ATP synthase subunit α is a binding partner for APP and Aβ on the surface of cultured hippocampal neurons and astrocytes indicating regulation of extracellular ATP levels in the brain. Human drusen were found to contain Aβ and this was interpreted as an indication that the pathogenic pathways giving rise to drusen and AMD may be common in neurodegenerative diseases characterized by misfolded protein aggregation [53]. San Li Xing et al., 2012 [41] showed in amyloid precursor protein/presennillin-1 transgenic mice that the α-subunit of ATP synthase is associated with aggregates of Aβ proteins in amyloid plaques and when extracellular ATP generation was analyzed a inhibition pattern was observed by the aggregating Aβ peptide but not the level of ATP synthase subunit alpha on neurons. Chronic exposure to soluble Aβ may result in an impairment of energy homeostasis due to a decreased respiratory capacity of mitochondrial electron transport chain which, in turn, may accelerate neurons demise [41].

We have addressed that Aβ is a pathological component in AD and AMD and that Aβ and APP can be addressed to the mitochondrion. In respect to new insights of the extramitochondrial role in energy production for eyesight [20,25] in the OS of rods and that Aβ directly binds to theα subunit of the ATP synthase at the neuronal membranes and the demonstration of a number of complexes to capture and direct electrons and protons in the cell, melatonin shows probably a primary constituent in balancing the energy production in mitochondrial by acting upon the production of Aβ. 

In the introduction we showed that melatonin regulates APP metabolsim and can efficiantly protect cells against Aβ toxicity, oxidative damage and cell death in vitro and in vivo [47]. A recent study showed that, chronic melatonin therapy in old Tg2576 mice initiated at 14 months of age failed to remove existing plaques, but also to prevent additional Aβ deposition [87]. Data on a diminished Aβ in melatonin-treated wild type mice [88] and reduced Aβ and protein nitration in melatonin treated Tg2576 mice also exist [89]. However, both studies concur in finding little evidence of the potent antioxidant effects of melatonin in the oldest mice. These findings indicate that melatonin has the ability to regulate APP metabolism and prevent Aβ pathology, but fails to exert anti-amyloid or antioxidant effects when initiated after the age of Aβ deposition. Although consistent conclusions were achieved, none of the related studies further explained how melatonin exerts its inhibitory effect on Aβ generation. One explanation of why aged mice are immune to melatonin might be in the process of melanogenesis, i.e. a failure in light/melanin/water system would be a cause rather than effect of AD has been proposed [90]. The decrease in melanins ability to dissociate water (human photosynthesis) in AMD [91] and or AD has been proposed to be a cause of these diseases is a simplistic overview of the bioenegetic mechanism related to these diseases. In our view hypometabolism, likely due to decline in both intra- and extra-mitochondrial OXPHOS functioning, are indeed fundamental to the understanding of pathological processes in these related diseases and that there is a homeostatic mechanism of energy balance related to relationship of melatonin *versus *Aβ through the regulation of mitochondrial fidelity. Melatonin protective role in AMD and AD may be a result of its action on mitochondrial physiology as suggested by its presence in mitochondrian circadian and seasonal variations in the brain and retina [92]. Locally produced melatonin in the surrounding of photoreceptors protects these cells thanks to its anti oxidant capacity or by activation of melatonin receptors [93]. Melatonin can increase membrane fluidity, as well as the activity of the ETC and ATP production, mitochondria membrane potential, while reducing oxidative stress [94]. Important pathological properties of Aβ, such as neurotoxicity and resistance to proteolytic degradation, depend on the ability of peptides to form β-sheet structures and/or amyloid fibrils [47]. Intervention in the Aβ aggregation process can be considered an approach to stopping or slowing the progression of AD and new investigation AMD. Melatonin can interact with Aβ40 and Aβ42 and inhibit the progressive formation of β-sheet and/or amyloid fibrils[95,96]. Melatonin could promote the conversion of β-sheets into random coils by disrupting the imidazole-carboxylate salt bridges and thus prevent Aβ fibrillogenesis and aggregation. It is therefore possible that by blocking the formation of the secondary β-sheet conformation, melatonin may not only reduce neurotoxicity but also facilitate clearance of the peptide via increased proteolytic degradation. 

However, it is difficult to determine the extent of the contribution from each of these properties to the overall effects of melatonin treatment in vivo. In mammals melatonin exerts some of its functions through two specific high-affinity membrane receptors belonging to the superfamily of G-protein-coupled receptors: MT1 and MT2. Decreased MT2 immunoreactivity and increased MT1 immunoreactivity have been reported in the hippocampus of AD patients [97]. Contrary to these findings, a study by Pappolla et al. [98] demonstrated that melatonin protective activities against Aβ toxicity does not require its binding to membrane receptors, which strongly suggests that protection is a result of its antioxidant and anti amyloidegenic features. Melatonin receptors have been found to modulate the visual function in mouse retina [99]. Numerous relationships are shown between melatonin and mitochondria in which protection of ETC proteins are crucial [94]. The hypothesis herein exposed has concentrated on the melatonin-Aβ axis in mitochondrial age related processes leading to AD and AMD. Still, there is a more complex view of this axis which is not in the scope of this paper, i.e. first, melatonin functions exceeds its role as hormone that mediates signal ‘’darkness’’, second melanocytes are viewed as ‘’neurons of the skin’’ with sensory and regulatory properties which can detect and transform external and internal signals/energy into organized regulatory networks for the maintenance of skin homeostasis [100] and melanogenesis and its product melanin is by itself an pigment that has extraordinary properties [101]. The most important property is melanin participation in electron transfer reactions, reducing and oxidizing other molecules. Also, its key monomer, indolequinone, exhibits photodriven proton transfer cycles [102]. Melanin has showed radiotropism, melanized fungi are stimulated to grow in environments with high ionizing radiation, suggesting melanin may function as a broad-band radiation energy harvester, similiar to chlorophyll [103].

In summary, the mitochondrion is the prime cross road enabling electron transfer for all these transfer, and it is reasonable that proton flow may represent a fundamental physical force that sustains, drives, and informs all biological organization and dynamics, Nevertheless, electron driven transport of protons would not be confined to the mitochondrion but it seems to be a fundamental properties of many cell membranes. 

## CONCLUSION

Both AMD and AD are age-related neurodegenerative diseases. They share similar environmental risk factors thereby comprising smoking, hypertension, hypercholesterolemia, atherosclerosis, obesity, and unhealthy diet [104]. The pathogenesis is associated with increased oxidative stress, and hypometabolism with impaired proteasomal and lysosomal function that evoke formation of intra- and extracellular deposits, drusen, lipofuscin and amyloid plaques, features of both AMD and AD, even though with a different genetic background. These facts imply a role for intra but also for extra-mitochondial OXPHOS.

We have addressed that Aβ is a pathological component in both AD and AMD and that both Aβ and APP can be addressed to the mitochondrion. Moreover, ATP synthase α-subunit was found to be a component of AMD drusen that in turn contain Aβ. New insights on the role of extramitochondrial energy production suggest that it may support visual process [5, 8] in the rod OS and neuronal conduction in myelin vesicles [23, 105] and are consistent with the finding that the α-subunit of ATP synthase is associated with Aβ in Alzheimer's disease [35]. Melatonin seems to be a primary constituent in balancing the energy production in mitochondrial by acting upon the production of Aβ. In fact, melatonin can regulate APP metabolism and efficiently protect cells against Aβ toxicity, oxidative damage and cell death, by interacting with Aβ40 and Aβ42 and inhibit the progressive formation of β-sheet and/or amyloid fibrils [47].

Our hypothesis does to some extent comprise an epigenetic paradigm coupling aging as an underling mechanism of AD and AMD.A genetic background would be a “blue print’’ in which environmental, genetic and bioenergetic factors (intra- and extra-mitochondrial energy production) tending to act upon them, thus leading to AMD and /or AD. There is a direct link between perturbed energy states in neurons and the retina [60] and creatinin and ATP metabolism. Also, there is a direct interaction between APP and the precursor of ubiquitous mitochondrial creatin kinase supporting a relationship between AD, cellular energy levels and mitochondrial function [106]. The same principle is allied to the retina and occurrence of AMD [60]. An understanding of the processes related to extra-mitochondrial and intra-mitochondrial regulation of metabolism in the brain and in retina and their balance by a melatonin-Aβ axis may emerge as new therapeutic pathway for the therapy of both AMD and AD. 

## DISCLOSURE

The authors report no conflicts of interest in this work.
